# Short-term efficacy of vedolizumab in patients with inflammatory bowel disease in real-life settings in Bosnia and Herzegovina

**DOI:** 10.17305/bb.2024.10433

**Published:** 2024-10-01

**Authors:** Nermin Salkić, Mirela Bašić Denjagić, Nađa Zubčević, Renata Tamburić, Azra Husić Selimović, Emil Babić, Milenko Bevanda, Aida Saray, Predrag Jovanović, Zoran Tošić, Aleksandar Dobrovoljski, Tatjana Barać

**Affiliations:** 1 Department of Internal Medicine, School of Medicine Tuzla, University of Tuzla, Tuzla, Bosnia and Herzegovina; 2 Department of Gastroenterology and Hepatology, University Clinical Center of Tuzla, Tuzla, Bosnia and Herzegovina; 3 Clinic of Gastroenterohepatology, University Clinical Center of Sarajevo, Sarajevo, Bosnia and Herzegovina; 4 Clinic of Gastroenterology and Hepatology, University Clinical Center of Republic of Srpska, Banja Luka, Bosnia and Herzegovina; 5 Department of Internal Medicine, General Hospital “Abdulah Nakaš” Sarajevo, Sarajevo, Bosnia and Herzegovina; 6 Department of Gastroenterology and Hepatology, University Clinical Hospital of Mostar, Mostar, Bosnia and Herzegovina; 7 Department of Gastroenterology, Health Center Brčko, Brčko, Bosnia and Herzegovina

**Keywords:** Inflammatory bowel disease (IBD), vedolizumab, Crohn’s disease (CD), ulcerative colitis (UC), real-world efficacy

## Abstract

Inflammatory bowel disease (IBD), encompassing Crohn’s disease (CD) and ulcerative colitis (UC), necessitates effective management strategies. This study aims to evaluate the real-world efficacy of vedolizumab, a newer biological therapy, in treating IBD in Bosnia and Herzegovina. A retrospective observational study was conducted across six medical centers, involving 139 IBD patients, 76 with UC and 63 with CD. Patients were assessed for clinical remission and other outcomes at the 26-week mark post vedolizumab treatment initiation. At 26 weeks, clinical remission was achieved in 82.9% of UC patients and 85.7% of CD patients. Mucosal healing was observed in 38.1% of CD patients. The efficacy of vedolizumab did not significantly differ based on prior anti-tumor necrosis factor (anti-TNF) exposure. Notably, the clinical scoring tools for predicting vedolizumab response showed limited applicability in this cohort. Vedolizumab demonstrated high efficacy in treating both UC and CD in real-world settings in Bosnia and Herzegovina, underscoring its potential as a significant therapeutic option in IBD management.

## Introduction

Inflammatory bowel disease (IBD), encompassing Crohn’s disease (CD) and ulcerative colitis (UC), is characterized by chronic inflammation of the gastrointestinal tract. The etiology of IBD is multifactorial, involving genetic, environmental, and immunological factors that contribute to an aberrant immune response in the gut mucosa [1]. The prevalence of IBD is rising globally, with recent data indicating a shift toward an increasing incidence in newly industrialized countries, including Bosnia and Herzegovina [2–5].

IBD presents significant therapeutic challenges in clinical practice. The management goals of IBD are to induce and maintain remission, improve quality of life, and prevent complications. Conventional therapies, such as corticosteroids and immunomodulators, have been the mainstay of treatment. However, their long-term use is often limited by side effects and decreasing efficacy over time [6].

With the emergence of biological agents, treatment paradigms for patients with moderate to severe IBD, who are either non-responsive or intolerant to conventional therapies, have evolved significantly. Initially, the therapeutic landscape for IBD was primarily dominated by anti-tumor necrosis factor (anti-TNF) agents. However, a notable proportion of patients either do not respond adequately to anti-TNF therapies, lose response over time to these treatments or must discontinue treatment due to adverse effects [7, 8].

In response to this unmet need, newer biological agents, such as vedolizumab, have been developed. Vedolizumab is a humanized monoclonal antibody that targets the α4β7 integrin on gut-homing lymphocytes, playing a crucial role in inhibiting leukocyte migration to the intestinal mucosa. This novel mechanism of action provides a valuable therapeutic alternative for patients with moderately to severely active IBD, particularly those who have had an inadequate response, lost response, or cannot tolerate conventional therapies or anti-TNF agents [9, 10].

The approval of vedolizumab by the European Medical Agency and the US Food and Drug Administration was supported by its efficacy in inducing and maintaining remission in patients with IBD, as demonstrated in several randomized controlled trials (RCTs) [9–12]. However, the real-world effectiveness of vedolizumab, particularly among diverse patient populations who might not meet the strict inclusion criteria of RCTs, remains an area of ongoing research [13].

In Bosnia and Herzegovina, the context presents unique challenges and opportunities for evaluating vedolizumab’s efficacy. Real-world data are essential for understanding the effectiveness of biologics in routine clinical settings, beyond the controlled environment of RCTs. Such data are instrumental in understanding patient responses in a more diverse population, including those typically excluded from clinical trials due to stringent inclusion and exclusion criteria. Real-world studies also provide insights into the drug’s impact on extra-intestinal manifestations of IBD and may reveal previously undetected safety signals [14].

Consequently, this study aims to assess the short-term efficacy of vedolizumab over a period of 26 weeks in the treatment of IBD in real-life clinical settings in Bosnia and Herzegovina. These insights may contribute significantly to the global understanding of the drug’s role in managing this complex disease.

## Materials and methods

### Patients

This retrospective analysis was conducted in Bosnia and Herzegovina, involving six medical centers: University Clinical Center of Tuzla, University Clinical Center of Banja Luka, University Clinical Center of Sarajevo, General Hospital “Abdulah Nakaš” Sarajevo, University Clinical Hospital of Mostar, and Health Center Brčko. The study was designed as a retrospective, observational, single-arm, multicenter study.

Eligible participants were individuals aged 18 years or older with active IBD at the initiation of vedolizumab treatment. Active CD was determined based on patient-reported outcome measures, coupled with one or more of the following: ulcers visible during colonoscopy, signs of active disease on magnetic resonance imaging (such as contrast enhancement, bowel thickening, or the comb sign), a C-reactive protein (CRP) level above the lower limit of detection, high-sensitivity CRP greater than 2.87 mg/L, or fecal calprotectin exceeding 200 µg/g, assessed not more than four weeks before treatment initiation. The threshold for CRP, as per most assays, was set at 4.0 mg/L. Active UC was defined by the presence of symptoms accompanied by a Mayo endoscopic subscore of 2 or higher, also evaluated within four weeks prior to the initiation of vedolizumab therapy [15].

Exclusion criteria were as follows: concurrent participation in any clinical trial where IBD treatment was part of an interventional study protocol, contraindications to vedolizumab such as known hypersensitivity to the drug or its excipients, prior exposure to vedolizumab, and planned cessation of treatment within 12 months from initiation (for instance, due to planned pregnancy) [16]. Patients who did not have all necessary albumin values on record, those who received therapy for pouchitis, or those treated for less than 26 weeks were also excluded.

The administration protocol for vedolizumab adhered to its summary of product characteristics: a 300 mg dose at weeks 0, 2, and 6, followed by a maintenance dose of 300 mg every eight weeks. For patients with CD who showed no response by week 10, an additional dose was permitted. However, given the retrospective nature of this study, the treating physician had the discretion to adjust the dose or dosing interval based on clinical judgment.

### Data collection

The data for this study were retrospectively collected from electronic hospital databases and written patient records. The cohort comprised patients diagnosed with CD and UC, treated across the six aforementioned centers in Bosnia and Herzegovina from January 2018 to June 2023.

For each patient, comprehensive medical records were reviewed to extract relevant data. This included demographic information (age, sex, and smoking status), disease characteristics (disease duration, location, and behavior in CD; extent of disease in UC), previous and concomitant IBD therapies, outcomes of these therapies (success or failure), exposure to anti-TNF drugs, previous surgeries, the presence of fistulizing disease in CD, and the details of vedolizumab treatment.

Disease activity was assessed at the initiation of vedolizumab treatment and at regular intervals thereafter, using standardized clinical indices: the Crohn’s Disease Activity Index (CDAI) for CD patients [17] and the Mayo score for UC patients [18]. Laboratory parameters, such as CRP and albumin levels, were recorded at baseline and after 26 weeks, if available.

If performed, endoscopic evaluations, radiologic imaging findings, and histopathological reports conducted as part of routine clinical care were also reviewed to corroborate clinical assessments of disease activity. The recorded data included values before and after 26 weeks of vedolizumab treatment. Additionally, we applied a recently described pretreatment clinical scoring tool for both CD and UC, which categorizes patients into three groups based on the probability of treatment response, thereby predicting the outcome of short-term treatment with vedolizumab [19, 20].

Data on clinical remission, response to therapy, and the need for surgical interventions were collected to assess the effectiveness of vedolizumab in this patient population. Due to the retrospective nature of the study, no reliable sources or methods were available to collect data on safety and adverse events; thus, these data were omitted from the analysis. All data were anonymized to ensure patient confidentiality.

### Outcomes

We specifically collected data on clinical remission and colectomy-free survival after 26 weeks for patients with UC. For patients with CD, data on clinical remission, steroid-free clinical remission, and mucosal healing were gathered after the same period.

**Table 1:** Baseline demographics and clinical characteristics of patients with Crohn’s disease and ulcerative colitis

	**Crohn’s disease (*n* ═ 76)**	**Ulcerative colitis (*n* ═ 63)**
Age, years, median (IQR)	39 (29–48)	42 (28–54)
Female sex, *n* (%)	29 (46.0)	36 (47.4)
Disease duration, years, median (IQR)	9 (4.5–13.5)	8 (4–11)
BMI, kg/m^2^, mean (SD)	23.0 (3.8)	24.4 (3.8)
CRP before treatment, mg/L, mean (SD)	22.0 (27.9)	19.2 (22.6)
Albumin before treatment, g/L, mean (SD)	35.0 (6.2)	34.2 (5.5)
Concomitant steroids, *n* (%)	42 (66.7)	56 (73.7)
Concomitant IMM, *n* (%)	36 (57.1)	47 (61.8)
Concomitant IMM and steroids, *n* (%)	29 (46.0)	41 (53.9)
Previous anti-TNF exposition, *n* (%)	41 (65.1)	39 (51.3)
Previous anti-TNF failure, *n* (%)	41 (65.1)	34 (44.8)
Extensive disease (UC only), *n* (%)		58 (76.4)
Previous surgery (CD only), *n* (%)	32 (50.8)	
Fistulizing disease (CD only), *n* (%)	14 (22.2)	
Localization (CD only), *n* (%)		
Ileocolonic	47 (74.6)	
Ileal	9 (14.3)	
Colonic	7 (11.1)	
Clinical scoring tool, *n* (%)		
Low probability of response	21 (33.3)	23 (30.3)
Intermediate probability of response	27 (42.9)	36 (47.4)
High probability of response	15 (23.8)	17 (22.4)

IQR: Interquartile range; BMI: Body mass index; SD: Standard deviation; CRP: C-reactive protein; IMM: Immunomodulators; anti-TNF: Anti-tumor necrosis factor; UC: Ulcerative colitis; CD: Crohn’s disease.

The detailed definition of the treatment goals in IBD can be found elsewhere [7, 8, 21], however, in general:

1. Clinical remission for CD was defined as a CDAI < 150 at week 26, consistent with the criteria used in the GEMINI 2 clinical trial [10];
2. Clinical remission for UC was defined by a full Mayo score of ≤ 2, with no subscore > 1, consistent with the criteria used in the GEMINI 1 clinical trial [9].

Steroid-free clinical remission was considered if the clinical remission was achieved and the patient had been off steroids for the 26-week period. Mucosal healing for CD was defined as either the absence of ulcers or erosions on ileocolonoscopy or the absence of inflammation indicators on cross-sectional imaging for patients who could not be adequately assessed with ileocolonoscopy, as defined in the VICTORY consortium study [22]. Colectomy-free survival was defined as the absence of a colectomy due to UC within the 26-week period.

For both UC and CD, clinical remission after 26 weeks was considered the primary outcome. Secondary outcomes included colectomy-free survival for UC and both steroid-free clinical remission and mucosal healing for CD after 26 weeks.

### Ethical statement

The study was conducted in accordance with the ethical guidelines of the Declaration of Helsinki. Data collection and analysis complied with local ethical guidelines and regulations. Given that the study utilized historical anonymized data, no institutional review board clearance was required.

### Statistical analysis

The baseline characteristics of the patients, including demographic data, disease characteristics, and previous treatments, were summarized using descriptive statistics. Continuous variables, such as age and duration of disease, were presented as means with standard deviations (SD) if normally distributed, or as medians with interquartile ranges (IQRs) in the case of non-normal distribution. Categorical variables, such as sex, smoking status, exposure to anti-TNF drugs, etc., were expressed as frequencies and percentages.

The effectiveness of vedolizumab was assessed by comparing clinical outcomes at the 26-week time point. The primary outcome measures were analyzed using the chi-square test or Fisher’s exact test for categorical variables. For continuous variables, changes over time were analyzed using paired *t*-tests or Wilcoxon signed-rank tests, depending on the data distribution. Subgroup analyses were conducted to explore differences in treatment response between various patient groups.

All statistical tests were two-sided, and a *P* value of less than 0.05 was considered to indicate statistical significance. Confidence intervals (95% CI) were calculated where appropriate. The data were analyzed using the open-source statistical software, JASP (Version 0.18.1).

## Results

We screened a total of 146 records and ultimately included 139 patients in the study by June 2023 – 76 (54.7%) with UC and 63 (45.3%) with CD. Of these, 22 (15.8%) patients were from the University Clinical Hospital of Mostar, 39 (28.1%) from the two Sarajevo centers combined, 55 (39.6%) from the University Clinical Center of Banja Luka, 16 (11.5%) from the University Clinical Center of Tuzla, and 7 (5.0%) from Health Center Brčko. The demographic and clinical characteristics of the study population are summarized in Table 1.

### Effectiveness of vedolizumab in CD

**Figure 1.**
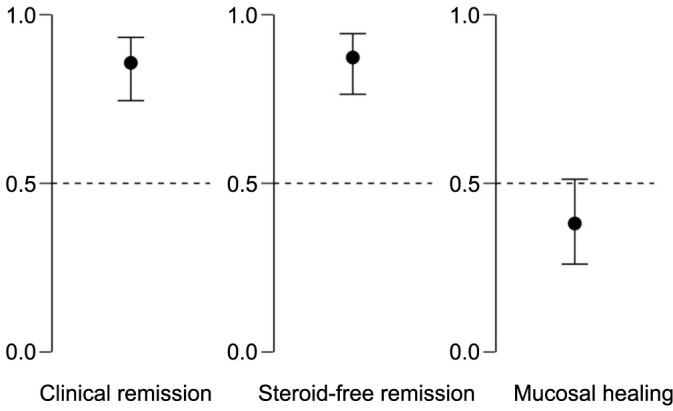
**Proportion (95% CI) of Crohn’s disease patients achieving primary and secondary outcomes after 26 weeks of treatment with vedolizumab.** CI: Confidence interval.

At the end of the 26-week treatment period, 54 out of the 63 patients (85.7%; 95% CI 74.6%–93.3%) achieved clinical remission as the primary outcome. As for secondary outcomes, steroid-free clinical remission was achieved in 55 out of the 63 patients (87.3%; 95% CI 76.5%–94.4%), while mucosal healing was achieved in 24 out of the 63 patients (38.1%; 95% CI 48.8%–73.9%) (Figure 1).

**Figure 2.**
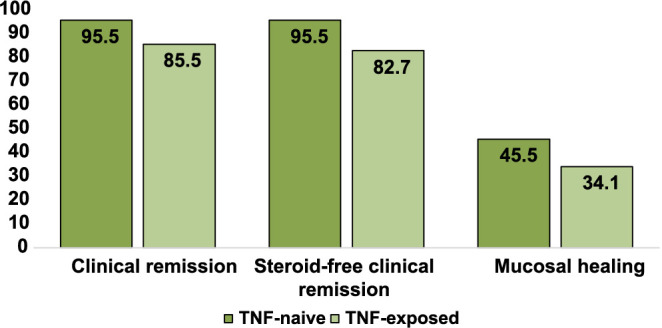
**Percentage of Crohn’s disease patients achieving primary and secondary outcomes after 26 weeks of treatment with vedolizumab, categorized by previous anti-TNF drug exposure.** anti-TNF: Anti-tumor necrosis factor.

Despite the fact that there was a clear tendency towards a better response in the subgroup of patients without previous anti-TNF exposure, this difference was not statistically significant (*P* > 0.05) (Figure 2).

### Effectiveness of vedolizumab in UC

**Figure 3.**
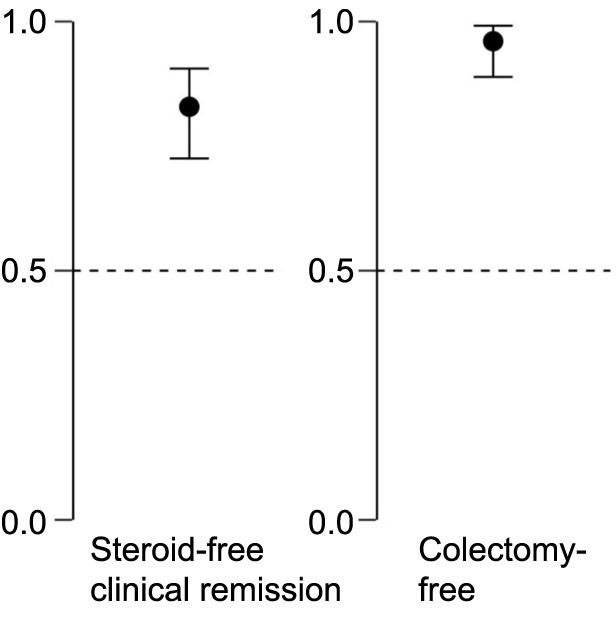
**Proportion (95% CI) of ulcerative colitis patients achieving primary and secondary outcomes after 26 weeks of treatment with vedolizumab.** CI: Confidence interval.

In total, 63 out of the 76 patients (82.9%; 95% CI 72.5%–90.6%) achieved steroid-free clinical remission as the primary outcome after the 26-week treatment period with vedolizumab. As for the secondary outcome, colectomy-free survival was achieved in 73 out of the 76 patients (96.1%; 95% CI 88.9%–99.2%) (Figure 3).

**Figure 4.**
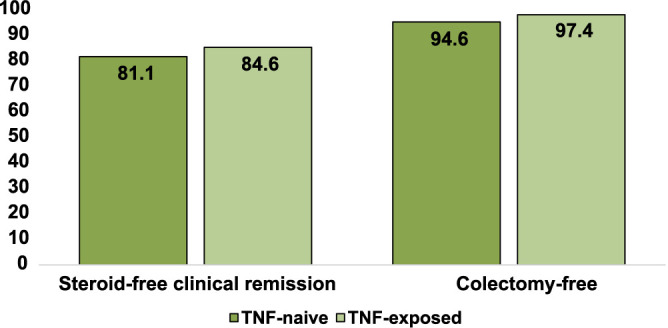
**Percentage of ulcerative colitis patients achieving primary and secondary outcomes after 26 weeks of treatment with vedolizumab, categorized by previous anti-TNF drug exposure.** anti-TNF: Anti-tumor necrosis factor.

No statistically significant difference in response to treatment was observed based on previous exposure to anti-TNF drugs (*P* > 0.05) (Figure 4).

### Predictive performance of clinical prediction tools for CD and UC

**Figure 5.**
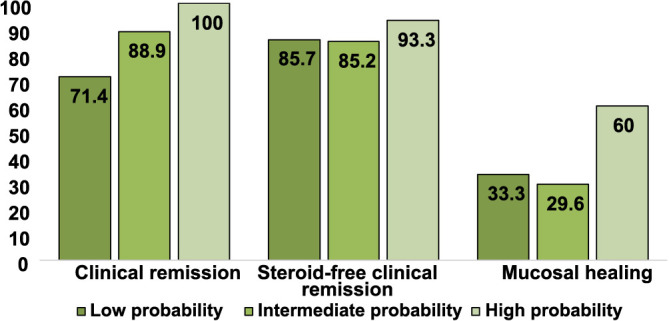
**Percentage of Crohn’s disease patients achieving primary and secondary outcomes after 26 weeks of treatment with vedolizumab, categorized by the clinical probability of response scoring tool described by Dulai et al.** **[20].**

We analyzed the proportion of CD patients achieving successful primary and secondary outcomes, based on the clinical prediction algorithm previously described, which categorizes patients into three groups according to their probability of treatment success [20]. The proportion of patients achieving successful outcomes based on the probability groups significantly differed only for clinical remission after 26 weeks (*P* ═ 0.045). However, there were no significant differences for steroid-free clinical remission and mucosal healing (*P* > 0.05) (Figure 5).

**Figure 6.**
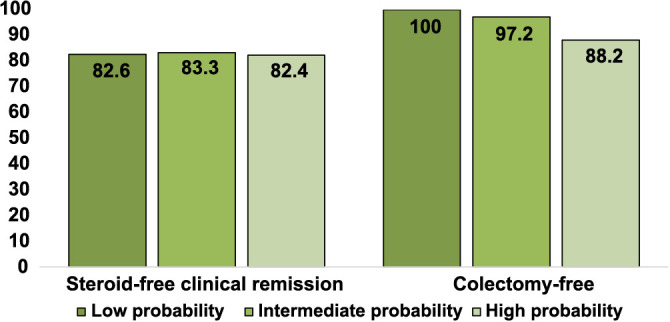
Percentage of ulcerative colitis patients achieving primary and secondary outcomes after 26 weeks of treatment with vedolizumab, categorized by the clinical probability of response scoring tool described by Dulai et al. [19].

Similarly, a clinical prediction tool that categorizes UC patients into three groups based on the probability of response was also previously described [19]. The analysis did not reveal any significant differences in the proportion of patients achieving a successful outcome according to these probability groups (*P* > 0.05) (Figure 6).

## Discussion

In this retrospective analysis, we assessed the short-term efficacy of vedolizumab in treating IBD in a real-life clinical setting in Bosnia and Herzegovina. To our knowledge, this is the first report of vedolizumab’s efficacy in real-world settings from this part of the world. Our findings revealed significant effectiveness in inducing clinical remission in both UC and CD within 26 weeks of treatment. This study contributes to the growing body of evidence supporting vedolizumab as a valuable treatment option for IBD management, particularly in real-world settings where patient populations are more diverse compared to those in RCTs.

Our results are in line with other real-world studies, which have generally reported favorable outcomes with vedolizumab in IBD treatment. However, our patients appear to have higher response rates in both CD and UC. For instance, an Italian multicenter study involving 729 patients (475 with UC and 254 with CD) reported a clinical remission at 6 months in 66.9% of patients, with 74.4% in CD and 62.9% in UC [22]. Over the follow-up period, they observed no significant difference in long-term remission rates between UC and CD (81.5% for both). Additionally, they reported a higher clinical response in UC patients (90.1%) compared to CD patients (84.3%). In contrast, our study found that 82.9% of UC patients and 85.7% of CD patients achieved clinical remission at 26 weeks, indicating a slightly different distribution of efficacy between UC and CD compared to the Italian study.

In comparing our study’s results with those from a German retrospective real-world study, we again observe notable differences in vedolizumab’s efficacy and safety [23]. The German study reported a 53.7% clinical remission rate in UC patients and a 14.4% clinical remission rate in CD patients treated with vedolizumab at 26 weeks, which contrasts with the higher remission rates in our study (82.9% in UC and 85.7% in CD at 26 weeks). Another German study similarly showed favorable outcomes after vedolizumab treatment, but again lower than in our cohort, with around a 50% response rate in both UC and CD [24]. Comparable outcomes were reported in a real-world study from the UK, where the response rates in UC and CD were around 45% [25].

A comparison of our findings with those from a meta-analysis incorporating real-world data also revealed higher rates of vedolizumab’s efficacy for IBD treatment [26]. The meta-analysis reported remission rates of 32% for UC and 30% for CD at week 14, which increased to 46% and 30%, respectively, at 12 months, whereas our study observed significantly higher remission rates at 26 weeks (82.9% for UC and 85.7% for CD).

Additionally, a study from Turkey showed response rates for UC (83%) and CD (50%) that were more aligned with our results, emphasizing regional/country-specific differences in response rates likely influenced by local availability, accessibility, and country-specific treatment algorithms [27].

This variability in vedolizumab’s effectiveness could be influenced by patient selection and treatment history, particularly in our study where vedolizumab was administered post-anti-TNF failure, underscoring its value as a salvage therapy in more resistant cases of IBD. These differences may also be attributable to various factors such as patient demographics, disease severity, and prior treatment history. Notably, while the Italian study observed a higher initial remission rate in CD, and the Turkish study reported higher rates in UC, our study found a more evenly distributed efficacy between UC and CD. Such variations highlight the importance of considering individual patient characteristics when evaluating treatment options.

It is also important to emphasize that the current treatment landscape in Bosnia and Herzegovina is not entirely favorable in terms of freely available treatment options. This limitation forces physicians to selectively choose patients who have the highest probability of responding to treatment. Results from the Epi IBD study in 2015 illustrate that patients in Eastern European countries are less likely to receive biologics and immunomodulators (14% and 54%, respectively) compared to patients in Western Europe (33% and 66%, respectively) [28]. The underperformance of the clinical scoring tools in our study could indeed be influenced by this selection bias. This careful selection process might have led to a patient cohort with distinct characteristics compared to those in the original Crohn’s Disease Scoring Tool (CDST) development study. Consequently, this could impact the tool’s performance and its applicability in our clinical setting, as the predictive parameters of the CDST may not align well with the characteristics of our selected patient population.

The high proportion of patients in our study who were previously exposed to anti-TNF agents is also noteworthy. It is well-established that patients who have failed anti-TNF therapy represent a particularly challenging group, often exhibiting more severe disease, limited treatment options, and potentially different responses to subsequent biological therapies [29, 30]. The use of vedolizumab in this context, often as a salvage therapy, underscores its role in managing complex IBD cases. Interestingly, despite the complexity and refractory nature of their disease, a substantial portion of these patients achieved clinical remission with vedolizumab, highlighting its efficacy even in a more treatment-resistant population. This success underscores both the efficacy of vedolizumab and the importance of proper patient selection, especially in settings with suboptimal treatment availability.

Our results, alongside those from other real-world studies, underline the importance of vedolizumab as a treatment option in the management of IBD. This is particularly relevant for patients who are either new to biologic therapies or have lost response to anti-TNF agents. Vedolizumab’s mechanism of action, targeting the α4β7 integrin, offers a therapeutic alternative, particularly for those with an inadequate response, lost response, or intolerance to conventional therapies or anti-TNF agents. Additionally, our results have significant implications for clinical practice in Bosnia and Herzegovina. They suggest that vedolizumab can be highly effective in treating IBD, even in patients who have previously failed anti-TNF therapies. This is particularly relevant given the increasing prevalence of IBD in the region and the challenges associated with managing complex cases [3–5].

It is of great importance to emphasize that the limited availability of treatments often forces physicians to reserve vedolizumab as a second-line treatment agent or salvage therapy. This pattern is clearly demonstrated in our cohort. Moving forward, it is essential that future studies address this issue by assessing the efficacy of vedolizumab as a first-line treatment. This is particularly significant in the case of UC, where the efficacy in anti-TNF naïve patients appears to be superior [31].

It is imperative to acknowledge the limitations inherent in retrospective studies and the need for ongoing research. Our study’s retrospective design introduces potential biases, particularly in patient selection and outcome reporting. Such biases may influence the observed high remission rates with vedolizumab, necessitating a cautious interpretation when generalizing these results. The retrospective nature also precluded reliable collection of safety data on the use of vedolizumab, which would have provided additional insights into not only the efficacy but also the safety of vedolizumab. Additionally, it is important to emphasize that there is no widely accepted and uniform corticosteroid tapering strategy in Bosnia and Herzegovina, leaving it to physician discretion. This variability means that some patients, depending on their disease history and status, may receive a longer or shorter course of steroids. Future studies from Bosnia and Herzegovina should aim to prospectively include larger and more diverse patient populations, enabling a more comprehensive understanding of vedolizumab’s role across different patient subgroups. Additionally, long-term studies are needed to assess the sustained efficacy and safety of vedolizumab, as well as its impact on health-related quality of life in IBD patients.

## Conclusion

In conclusion, our study contributes valuable evidence supporting the efficacy of vedolizumab in the treatment of IBD within the real-world clinical settings of a country with limited healthcare resources, such as Bosnia and Herzegovina. Our findings align with those from other real-world studies, underscoring vedolizumab’s ability to induce and maintain remission in both UC and CD. These results reinforce the role of vedolizumab as an effective therapeutic option in the IBD treatment landscape, offering hope for patients with this complex and challenging condition.

## References

[1] Baumgart DC, Sandborn WJ (2007). Inflammatory bowel disease: clinical aspects and established and evolving therapies. Lancet.

[2] Ng SC, Shi HY, Hamidi N, Underwood FE, Tang W, Benchimol EI (2017). Worldwide incidence and prevalence of inflammatory bowel disease in the 21st century: a systematic review of population-based studies. Lancet.

[3] Salkic NN, Pavlovic-Calic N, Gegic A, Jovanovic P, Basic M (2010). Ulcerative colitis in the Tuzla region of Bosnia and Herzegovina between 1995 and 2006: epidemiological and clinical characteristics. Eur J Gastroenterol Hepatol.

[4] Pavlovic-Calic N, Salkic NN, Gegic A, Smajic M, Alibegovic E (2008). Crohn’s disease in Tuzla region of Bosnia and Herzegovina: a 12-year study (1995–2006). Int J Colorectal Dis.

[5] Tulumović E, Salkić N, Tulumović D (2021). Inflammatory bowel disease in Tuzla Canton, Bosnia-Herzegovina: a prospective 10-year follow-up. World J Gastroenterol.

[6] Hanauer SB, Feagan BG, Lichtenstein GR, Mayer LF, Schreiber S, Colombel JF (2002). Maintenance infliximab for Crohn’s disease: the ACCENT I randomised trial. Lancet.

[7] Raine T, Bonovas S, Burisch J, Kucharzik T, Adamina M, Annese V (2022). ECCO guidelines on therapeutics in ulcerative colitis: medical treatment. J Crohns Colitis.

[8] Torres J, Bonovas S, Doherty G, Kucharzik T, Gisbert JP, Raine T (2020). ECCO guidelines on therapeutics in Crohn’s disease: medical treatment. J Crohns Colitis.

[9] Feagan BG, Rutgeerts P, Sands BE, Hanauer S, Colombel J-F, Sandborn WJ (2013). Vedolizumab as induction and maintenance therapy for ulcerative colitis. N Engl J Med.

[10] Sandborn WJ, Feagan BG, Rutgeerts P, Hanauer S, Colombel J-F, Sands BE (2013). Vedolizumab as induction and maintenance therapy for Crohn’s disease. N Engl J Med.

[11] Attauabi M, Madsen GR, Bendtsen F, Seidelin JB, Burisch J (2022). Vedolizumab as the first line of biologic therapy for ulcerative colitis and Crohn’s disease—a systematic review with meta-analysis. Digest Liver Dis.

[12] Feagan BG, Schreiber S, Wolf DC, Axler JL, Kaviya A, James A (2019). Sustained clinical remission with Vedolizumab in patients with moderate-to-severe ulcerative colitis. Inflammat Bowel Dis.

[13] Colombel JF, Sandborn WJ, Reinisch W, Mantzaris GJ, Kornbluth A, Rachmilewitz D (2010). Infliximab, Azathioprine, or combination therapy for Crohn’s disease. N Engl J Med.

[14] Chen D (2022). Real-world studies: bridging the gap between trial-assessed efficacy and routine care. J Biomed Res.

[15] Kucharzik T, Ellul P, Greuter T, Rahier JF, Verstockt B, Abreu C (2021). ECCO guidelines on the prevention, diagnosis, and management of infections in inflammatory bowel disease. J Crohns Colitis.

[16] Eriksson C, Rundquist S, Lykiardopoulos V, Udumyan R, Karlén P, Grip O (2021). Real-world effectiveness of Vedolizumab in inflammatory bowel disease: week 52 results from the Swedish prospective multicentre SVEAH study. Therap Adv Gastroenterol.

[17] Best WR, Becktel JM, Singleton JW, Kern F (1976). Development of a Crohn’s disease activity index. Gastroenterology.

[18] Lewis JD, Chuai S, Nessel L, Lichtenstein GR, Aberra FN, Ellenberg JH (2008). Use of the noninvasive components of the mayo score to assess clinical response in ulcerative colitis. Inflammat Bowel Dis.

[19] Dulai PS, Singh S, Vande Casteele N, Meserve J, Winters A, Chablaney S (2020). Development and validation of clinical scoring tool to predict outcomes of treatment with Vedolizumab in patients with ulcerative colitis. Clin Gastroenterol Hepatol.

[20] Dulai PS, Boland BS, Singh S, Chaudrey K, Koliani-Pace JL, Kochhar G (2018). Development and validation of a scoring system to predict outcomes of Vedolizumab treatment in patients with Crohn’s disease. Gastroenterology.

[21] Le Berre C, Ricciuto A, Peyrin-Biroulet L, Turner D (2022). Evolving short- and long-term goals of management of inflammatory bowel diseases: getting it right, making it last. Gastroenterology.

[22] Dulai PS, Singh S, Jiang X, Peerani F, Narula N, Chaudrey K (2016). The Real-world effectiveness and safety of Vedolizumab for moderate–severe Crohn’s disease: results from the US VICTORY consortium. Amer J Gastroenterol.

[23] Mocci G, Tursi A, Maconi G, Cataletti G, Mantia B, Serio M (2023). Real-world efficacy and safety of Vedolizumab in managing ulcerative colitis versus Crohn’s disease: results from an Italian multicenter study. Expert Opin Biol Ther.

[24] Helwig U, Mross M, Schubert S, Hartmann H, Brandes A, Stein D (2020). Real-world clinical effectiveness and safety of Vedolizumab and anti-tumor necrosis factor alpha treatment in ulcerative colitis and Crohn’s disease patients: a German retrospective chart review. BMC Gastroenterol.

[25] Hoffmann P, Krisam J, Stremmel W, Gauss A (2019). Real-world outcomes of Vedolizumab therapy in ulcerative colitis and Crohn’s disease at a tertiary referral center. Dig Dis.

[26] White JR, Din S, Ingram RJM, Foley S, Alam MA, Robinson R (2020). Experiences of using Vedolizumab in the treatment of inflammatory bowel disease in the East Midlands UK—a retrospective observational study. Scand J Gastroenterol.

[27] Schreiber S, Dignass A, Peyrin-Biroulet L, Hather G, Demuth D, Mosli M (2018). Systematic review with meta-analysis: real-world effectiveness and safety of vedolizumab in patients with inflammatory bowel disease. J Gastroenterol.

[28] Erdoğan Ç, Yeşil B, Bacaksız F, Arı D, Gökbulut V, Yüksel M (2022). Use of Vedolizumab in inflammatory bowel disease: a single-center experience. Turk J Gastroenterol.

[29] Burisch J, Kiudelis G, Kupcinskas L, Kievit HAL, Andersen KW, Andersen V (2019). Natural disease course of Crohn’s disease during the first 5 years after diagnosis in a European population-based inception cohort: an Epi-IBD study. Gut.

[30] Feagan BG, Lasch K, Lissoos T, Cao C, Wojtowicz AM, Khalid JM (2019). Rapid response to Vedolizumab therapy in biologic-Naive patients with inflammatory bowel diseases. Clin Gastroenterol Hepatol.

[31] Bressler B, Yarur A, Silverberg MS, Bassel M, Bellaguarda E, Fourment C (2021). Vedolizumab and anti-tumour necrosis factor α real-world outcomes in biologic-naïve inflammatory bowel disease patients: results from the EVOLVE study. J Crohn’s Colitis.

